# Improving the Rechargeable Li‐CO_2_ Battery Performances by Tailoring Oxygen Defects on Li‐Ni‐Co‐Mn Multi‐Metal Oxide Catalysts Recycled from Spent Ternary Lithium‐Ion Batteries

**DOI:** 10.1002/advs.202402892

**Published:** 2024-05-17

**Authors:** Juan Wang, Ningning Feng, Shuang Zhang, Yang Lin, Yapeng Zhang, Jing Du, Senlin Tian, Qun Zhao, Gang Yang

**Affiliations:** ^1^ Kunming University of Science and Technology Kunming 650093 P. R. China; ^2^ Suzhou Key Laboratory of Functional Ceramic Materials Department Changshu Institute of Technology Suzhou 215500 P. R. China

**Keywords:** cathodic catalysts, Li‐CO_2_ batteries, multi‐element metal oxides, oxygen vacancies, spent LiNi_0.8_Co_0.1_Mn_0.1_O_2_ cathodes

## Abstract

Rechargeable Li‐CO_2_ batteries are considered as a promising carbon‐neutral energy storage technology owing to their ultra‐high energy density and efficient CO_2_ capture capability. However, the sluggish CO_2_ reduction/evolution kinetics impedes their practical application, which leads to huge overpotentials and poor cyclability. Multi‐element transit metal oxides (TMOs) are demonstrated as effective cathodic catalysts for Li‐CO_2_ batteries. But there are no reports on the integration of defect engineering on multi‐element TMOs. Herein, the oxygen vacancy‐bearing Li‐Ni‐Co‐Mn multi‐oxide (Re‐NCM‐H3) catalyst with the *α*‐NaFeO_2_‐type structure is first fabricated by annealing the NiCoMn precursor that derived from spent ternary LiNi_0.8_Co_0.1_Mn_0.1_O_2_ cathode, in H_2_ at 300 °C. As demonstrated by experimental results and theory calculations, the introduction of moderate oxygen vacancy has optimized electronic state near the Fermi level (E_f_), eventually improving CO_2_ adsorption and charge transfer. Therefore, the Li‐CO_2_ batteries with Re‐NCM‐H3 catalyst deliver a high capacity (11808.9 mAh g^−1^), a lower overpotential (1.54 V), as well as excellent stability over 216 cycles at 100 mA g^−1^ and 165 cycles at 400 mA g^−1^. This study not only opens up a sustainable application of spent ternary cathode, but also validates the potential of multi‐element TMO catalysts with oxygen defects for high‐efficiency Li‐CO_2_ batteries.

## Graphical abstract

The Li‐Ni‐Co‐Mn multi‐element metal oxide (Re‐NCM‐H3) catalyst with moderate oxygen vacancies is synthesized by recycling from spent ternary NCM cathode and using an annealing approach. Owing to double characteristics of muti‐metal active sites and oxygen vacancies, the Li‐CO_2_ battery based Re‐NCM‐H3 catalyst shows an excellent stability over 216 cycles and a lower overpotential of 1.54 V at 100 mA g^−1^.

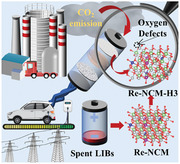

## 1 Introduction

Due to the excessive exploitation and utilization of fossil fuels, the increasing atmospheric CO_2_ concentration has led to the inevitable greenhouse effect and irreversible climate change.^[^ ^1^, ^2^ ^]^ Thus, over the past decades, substantial efforts have been devoted to designing multiple technologies for capturing superfluous CO_2_ and turning it into high‐value chemicals or fuels. Among these carbon‐neutral technologies, rechargeable Li‐CO_2_ batteries are regarded as one of the most promising means to both electrochemically capture CO_2_ and efficiently store electrical energy.^[^ ^3^, ^4^ ^]^ In 2014, Liu et al.^[^ ^5^ ^]^ first found that the true Li‐CO_2_ battery could be reversibly cycled on the basis of the electrochemical reaction of 4Li + 3CO_2_ ↔ 2Li_2_CO_3_ + C (*E_0_* = 2.8 V vs Li^+^/Li). Accordingly, this attractive battery system can deliver the high theoretical energy density of 1876 Wh kg^−1^,^[^ ^6^ ^]^ which evokes the researchers’ enthusiasm with potential for electrical vehicles. Moreover, Li‐CO_2_ batteries possess the potential of power utilization for submarine operation and Mars exploration with high CO_2_ content.^[^ ^1^ ^]^ However, the practical application of Li‐CO_2_ batteries has been impeded by many formidable challenges, such as huge discharge/charge overpotentials, poor rate capability, inferior cycle stability and even aggressive electrolyte decomposition. These originate from the sluggish kinetics of CO_2_ reduction reaction (CO_2_RR) and CO_2_ evolution reaction (CO_2_ER) as a consequence of the insulating characteristic of Li_2_CO_3_ product with wide band‐gap.^[^ ^7^, ^8^, ^9^, ^10^ ^]^ Hence, there is an urgent need to solve these above tricky problems to accelerate the rechargeable Li‐CO_2_ batteries toward real applications.

Fortunately, a lot of researches have been dedicated to designing highly effective cathodic catalysts to promote the electrochemical kinetics of CO_2_RR and CO_2_ER for the high‐performance Li‐CO_2_ batteries. Among the cathodic catalysts, heterogenous catalysts have been intensively studied, including carbon‐based materials, noble metals, transition metal oxides or carbides and so forth.^[^ ^11^, ^12^, ^13^, ^14^, ^15^, ^16^, ^17^ ^]^ Generally, carbon‐based materials, employed as cathodic catalysts for rechargeable Li‐CO_2_ batteries, have exhibited pretty good CO_2_RR catalytic activity due to their large specific surface area and superior electron conductivity.^[^ ^18^ ^]^ However, carbon materials have a poor catalytic effect on the decomposition of Li_2_CO_3_ during charging. In terms of noble metals, they have been certified to remarkably reduce the charge overpotential and improve the energy efficiency and cycling stability in Li‐CO_2_ batteries.^[^ ^19^ ^]^ But there is no possibility of large‐scale application of noble metals as electrocatalysts due to their huge cost and scarcity. Thereby, transition metal oxides (TMOs) have attracted more attention because of their considerable catalytic activity, relatively high stability and low cost. Ma et al.^[^ ^20^ ^]^ reported porous Mn_2_O_3_ cathodic catalyst for Li‐CO_2_ batteries. And the corresponding Li‐CO_2_ battery delivered a reduced discharge/charge voltage gap of ≈1.4 V and prolonged cycling life (2000 h) at a current density of 50 mA g^−1^. Nevertheless, the utilization of unitary TMOs has been severely obstructed by the limited catalytic sites and poor intrinsic conductivity.^[^ ^21^ ^]^ To overcome these bottlenecks, two new strategies have been put forward recently to improve the electrocatalytic activity of unitary TMOs. On the one hand, the doping optimization of foreign metal into single TMO catalyst has been identified as a reasonable approach to improve the electronic conductivity of multi‐metal oxide catalysts in the domain of Li–O_2_ batteries,^[^ ^22^ ^]^ hydrogen‐evolving and oxygen‐evolving reaction (HER and OER),^[^ ^23^ ^]^ as well as Li‐CO_2_ batteries.^[^ ^24^, ^25^ ^]^ By virtue of the synergistic effect between different metallic elements and multiple active sites for CO_2_ adsorption, this kind of multi‐metal oxides are proposed to promote the electrocatalytic kinetics of CO_2_RR and CO_2_ER. For example, Ge et al.^[^ ^24^ ^]^ designed the Co‐doped *α*‐MnO_2_ nanowire catalyst Co_0.2_Mn_0.8_O_2_ for rechargeable Li‐CO_2_ batteries, which can deliver a high capacity of 8160 mAh g^−1^, a reduced overpotential of ≈0.73 V and an extended cycling lifetime of over 500 cycles at a current density of 100 mA g^−1^. Xiao et al.^[^ ^25^ ^]^ successfully prepared a binary metal oxide Co_0.1_Ni_0.9_O_x_/CNT as the cathode, in which rechargeable Li‐CO_2_ batteries operated for 50 cycles without capacity degradation at a current density of 100 mA g^−1^ under a limitative capacity of 500 mAh g^−1^. On the other hand, the modulation of vacancy defects on TMOs is another desirable method to regulate the electronic structure and conductivity of TMOs, and thus boosting the kinetics for electrochemical CO_2_ conversion. For instance, the oxygen vacancy‐rich NiO nanosheets on carbon cloth has been obtained as a high‐efficiency electrocatalyst for Li‐CO_2_ batteries with a lower discharge–charge overpotential and superior cycle stability.^[^ ^26^ ^]^ Therefore, it is expected that the integration of oxygen defects and multi‐metal oxide catalysts could improve their conductivity and catalytic behaviors in Li‐CO_2_ batteries.

Recent works demonstrate that reusable multi‐metal oxide electrocatalysts, derived from the spent Li‐ion battery cathode materials, have been successfully applied in the field of Zn‐air batteries,^[^ ^27^ ^]^ Li–O_2_ batteries,^[^ ^28^ ^]^ Li–S batteries,^[^ ^29^ ^]^ organic pollutants degradation,^[^ ^30^, ^31^ ^]^ OER^[^ ^32^ ^]^ and CO_2_ reduction.^[^ ^33^ ^]^ For example, Bian et al.^[^ ^32^ ^]^ converted spent LiCoO_2_ cathode to an effective Co_9_S_8_/Co_3_O_4_ catalyst for OER by a conventional hydrometallurgy and sulfidation process, and the overpotential at 10 mA cm^−2^ was 274 mV and Tafel slope was 48.7 mV dec^−1^. Jiao et al.^[^ ^27^ ^]^ recycled NiMnCo‐activated carbon with core‐shell structure from spent LiNi_1‐x‐y_Co_x_Mn_y_O_2_ (NCM) cathode, a bifunctional catalyst for Zn‐air batteries, which possessed a lower voltage gap of 0.72 V at the initial three cycles and long cycle lifespan of 200 h at 10 mA cm^−2^. The layer‐structured ternary NCM cathodes, rich in valuable metals (such as Li, Co, Mn, and Ni), has dominated the current market because of their attractive energy density. Recently, our group introduced Li_x_MO (M = Ni/Co/Mn) with different lithium contents from spent NCM as cathodic catalysts for Li–O_2_ battery to alleviate the discharge/charge polarization and ameliorate cyclic stability.^[^ ^28^ ^]^ Additionally, according to the statistics, China's output of retired lithium‐ion batteries (LIBs) reached 12.9 GWh in 2020, and will extend to 117 GWh in 2025.^[^ ^34^ ^]^ This data indicates that low‐cost multi‐TMO electrocatalysts from retired LIBs could be applied on a large scale in the near future. Nevertheless, these multi‐metal oxides as electrocatalysts, recycled directly from spent LIBs, have still suffered from some barriers, such as less catalytic active sites, poor conductivity and large particle size. To our best knowledge, the employment of multi‐TMO‐based catalysts, derived from spent ternary cathode materials, has not been investigated in the rechargeable Li‐CO_2_ batteries until now. Inspired by these aforementioned facts, fabricating oxygen vacancy on multi‐metal oxides from spent NCM materials must be a feasible method to update the rechargeable Li‐CO_2_ battery performance.

Herein, we used a Li‐Ni‐Co‐Mn multi‐metal oxide, recovered from the spent NCM cathode materials by a simple sintering process, as the precursor to evaluate systemically the role of oxygen vacancies on catalytic activity in rechargeable Li‐CO_2_ batteries. First, we investigated the influence of the annealing temperature on oxygen vacancies in the recycled Li‐Ni‐Co‐Mn multi‐metal oxide. And the oxygen vacancy‐bearing Li‐Ni‐Co‐Mn multi‐metal oxide catalyst kept with the *α*‐NaFeO_2_‐type layered structure was obtained by annealing in H_2_ atmosphere at the optimum temperature of 300 °C, which was denoted as Re‐NCM‐H3. During the thermal reduction process, the O atoms were removed from the Li‐Ni‐Co‐Mn oxide matrix to generate oxygen vacancies and unpaired electrons were redistributed to adjacent metal atoms subsequently, thus facilitating the adsorption and charge transfer during CO_2_RR. Density functional theory (DFT) calculations also identified that the presence of moderate oxygen vacancies on the surface of Li‐Ni‐Co‐Mn multi‐oxide catalyst can regulate the electronic states close to the Fermi level (E_f_), resulting in strengthening the electronic conductivity and intrinsic catalytic activity of Re‐NCM‐H3. Moreover, the reduced particle size of Re‐NCM‐H3 catalyst after H_2_‐annealing entailed large surface area, thereby providing more catalytic active sites for CO_2_RR or/and CO_2_ER. Benefitting from the synergistic interaction of multi‐metal active sites and more oxygen vacancies, the assembled Li‐CO_2_ battery with oxygen vacancy‐bearing Li‐Ni‐Co‐Mn multi‐oxide cathodic catalyst delivered a lower overpotential and excellent cycle stability. Besides, this work can assist the rational design of upcycled multi‐TMO electrocatalysts from spent LIBs to Li‐CO_2_ batteries via defect engineering.

## 2 Results and Discussion

### 2.1 Controllable Production of Oxygen Vacancies on Re‐NCM Recovered from Spent LIBs

**Figure 1:**
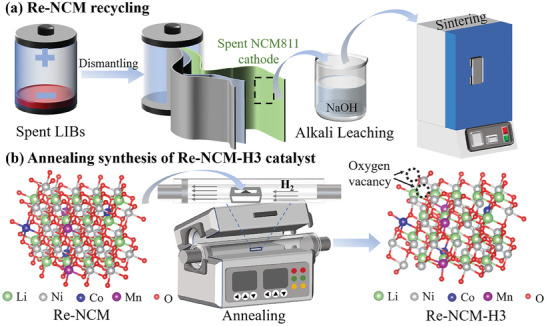
Schematic illustrations of the fabrication process of the oxygen vacancy‐bearing Li‐Ni‐Co‐Mn oxide (Re‐NCM‐H3) catalyst from spent LIBs. a) Recovery of the NiCoMn precursor (Re‐NCM) from spent LIBs. A mixed NiCoMn solution was obtained by dissolving a spent NCM811 cathode in sodium hydroxide. Then, the NiCoMn precipitate was sintered to prepare the Re‐NCM precursor. b) Schematic of the H_2_‐annealing method to synthesize the oxygen vacancies‐rich Re‐NCM‐H3 catalyst continuously.

**Figure 2:**
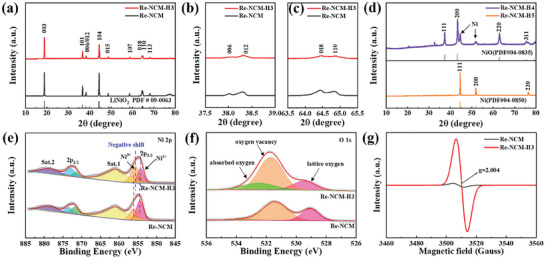
a,b) XRD patterns of Re‐NCM and Re‐NCM‐H3. Enlarged views of the (b) (006)/(102) peaks and c) (108)/(110) peaks. d) XRD patterns of Re‐NCM‐H4 and Re‐NCM‐H5. e) The Ni 2p and (d) O1s XPS spectra of Re‐NCM and Re‐NCM‐H3. f) EPR spectra of Re‐NCM and Re‐NCM‐H3.

The oxygen vacancy‐bearing Li‐Ni‐Co‐Mn multi‐metal oxide catalyst was synthesized by the recycling of NiCoMn precursor from a spent LiNi_0.8_Co_0.1_Mn_0.1_O_2_ (NCM811) cathode and a following hydrogen annealing process, as illustrated in **Figure** **1**. The NiCoMn precursor (referred as Re‐NCM) was obtained by air‐sintering of the spent NCM811 cathode. Then the series of Re‐NCM‐based catalysts rich in oxygen vacancies were gained by annealing at 300, 400, 500 °C, referring as Re‐NCM‐H3, Re‐NCM‐H4, Re‐NCM‐H5, respectively. Detailed synthetic processes are shown in the Experimental Section. X‐ray diffraction (XRD) was adopted to explore the effect of various reduction temperatures on the changed microstructure of the prepared catalysts. As shown in **Figure** **2**a, both Re‐NCM‐H3 and Re‐NCM maintain the *α*‐NaFeO_2_‐type layered structure (*R* $\bar{3}$ *m* space group) without other detectable impurity peaks. However, for these two samples, the indefinite splitting of the peaks (006)/(012) and (018)/(110) shows their low crystallinity and lithium deficiency (Figure 2b,c).^[^ ^35^ ^]^ Moreover, the intensity ratio of I_(003)_/I_(104)_ of Re‐NCM and Re‐NCM‐H3 materials are lower than 1.2 (1.012 and 1.150, respectively), indicating the certain degree of Li^+^/Ni^2+^ cation disorder of two samples. As reported in recent literature, the cation mixing can stabilize the layered structure at the de‐lithiated NCM significantly.^[^ ^36^ ^]^ By contrast, after annealed at 400 °C in 5% H_2_/Ar, a dual system comprising of cubic NiO phase (PDF 04‐0835) and metallic Ni (PDF 04‐0850) can be observed from the XRD pattern of Re‐NCM‐H4 in Figure 2d. Even in 5% H_2_/Ar reduction at 500 °C, the characteristic peaks of the Re‐NCM‐H5 catalyst at 44.5°, 51.8° and 76.3° can match well with (111), (200), and (220) planes of metallic Ni (PDF 04‐0850), respectively. The corresponding Ni 2p spectra of Re‐NCM‐H4 and Re‐NCM‐H5 are in a good agreement with their XRD result, respectively (shown in Figure S1, Supporting Information). These results have indicated that the higher H_2_‐treatment temperature can destroy the *α*‐NaFeO_2_‐type layered structure of Re‐NCM catalyst and generate new phase. Therefore, the determination of the optimal annealing temperature at 300 °C is a critical issue to retain the layered structure without phase transition while designing the experimental process. As Table S1 (Supporting Information) lists, the elemental composition of Re‐NCM and Re‐NCM‐H3 were obtained by inductively coupled plasma optical emission spectrometry (ICP‐OES). The molar ratios of Li, Ni, Co, and Mn in these two samples were calculated to remain nearly constant. Notably, the content of O in Re‐NCM‐H3 is lower than that of Re‐NCM, which is ascribed to the fact that the H_2_‐annealing treatment is more likely to produce oxygen vacancies.

To further probe the change of surface oxidation state of the catalysts, the X‐ray photoelectron spectroscopy (XPS) measurement was measured on Re‐NCM and Re‐NCM‐H3. As depicted in Figure 2e, Ni 2p spectrum of both two samples can be classified into two satellite peaks at 861.2 and 879.0 eV, which are assigned to Ni 2p_3/2_ and Ni 2p_1/2_, respectively. The peaks located at 856.1 and 873.4 eV can be distributed to Ni^3+^, and other signal peaks situated at 854.4 and 871.7 eV belong to Ni^2+^.^[^ ^37^ ^]^ In addition, quantitative analysis of Ni 2p spectra has revealed that the proportion of Ni^3+^ in the Re‐NCM‐H3 sample was markedly increased to maintain the charge balance after H_2_ annealing treatment, which corresponds to the generation of oxygen vacancies.^[^ ^38^ ^]^ This result agrees well with the lower level of Li^+^/Ni^2+^ disorder for Re‐NCM‐H3 acquired from the above XRD analysis. Apart from the change in the integrated peak area of Ni^3+^/Ni^2+^ ratio, the negative shift of Ni 2p binding energy in the Re‐NCM‐H3 sample has also signified the existence of oxygen vacancy defects.^[^ ^38^, ^39^ ^]^ This result can be further confirmed by the O 1s spectra. As exhibited in Figure 2f, the O 1s XPS spectra of Re‐NCM and Re‐NCM‐H3 can be all deconvoluted into three peaks, including the lattice oxygen of metal‐oxygen, oxygen vacancy, and adsorbed oxygen, centered at 529.4, 531.7, and 532.5 eV, respectively.^[^ ^26^ ^]^ It should be noted that the peak area contribution of oxygen defects and adsorbed oxygen for Re‐NCM is relatively low, indicating less concentration of oxygen vacancies in the host Re‐NCM sample. Nevertheless, after being reduced by H_2_ annealing, not only the intensity of oxygen vacancy peak is significantly enhanced (oxygen vacancy peak ratio in the XPS results of Table S2 (Supporting Information) increased from 20.03% for Re‐NCM to 32.49% for Re‐NCM‐H3), but also the intensity of lattice oxygen peak is weakened (lattice oxygen peak ratio in the XPS results of Table S2 (Supporting Information) decreased from 29.97% for Re‐NCM to 17.51% for Re‐NCM‐H3). Meanwhile, the contribution from adsorbed oxygen for Re‐NCM‐H3 is obviously larger than that for Re‐NCM sample, indicating the increasing of oxygen vacancies for active sites to bond oxygen. Additionally, the valence states of other active metal elements (Co and Mn) are also studied by using the Co 2p and Mn 2p spectra (Figure S2, Supporting Information). As the previous literature reported, the Co 2p spectrum of these two samples can be divided into two characteristic peaks at 780.08 and 794.88 eV, corresponding to Co 2p_3/2_ and Co 2p_1/2_, respectively.^[^ ^37^ ^]^ And the peaks located at 780.06 and 795.25 eV can be assigned to Co^3+^ and another signal peaks situated at 782.9 and 798.7 eV belong to Co^2+^. Quantitative analysis of Co 2p spectra has indicated that the value of Co^3+^/Co^2+^ decreases from 3.22 for Re‐NCM to 2.93 for Re‐NCM‐H3, further confirming the increasing density of oxygen vacancies and the reduction of Co^3+^ to Co^2+^ after H_2_‐annealing treatment. Similar phenomenon also appears in their Mn 2p XPS spectra. Both Re‐NCM and Re‐NCM‐H3 samples have two spin‐orbit doublets of Mn 2p_3/2_ at 642.3 eV and Mn 2p_1/2_ at 653.9 eV and the satellite peaks. And these peaks can be deconvoluted into Mn^4+^ (at 644.5 and 654.8 eV) and Mn^3+^ (at 643.3 and 653.2 eV), respectively.^[^ ^37^ ^]^ According to the previous study, the lower Mn^4+^/ Mn^3+^ ratio generally indicates more oxygen vacancies. Figure S2 (Supporting Information) shows that the ratio of Mn^4+^/ Mn^3+^ also cuts down from 1.0 for Re‐NCM to 0.61 for Re‐NCM‐H3, indicating that the conversion of some Mn^4+^ ions into Mn^3+^ accompanies with the generation of oxygen vacancies. Therefore, these XPS results have demonstrated that the H_2_‐annealing procedure can induce the creating of more oxygen vacancies in the multi‐metal oxides. It is widely believed that the formation of oxygen vacancies provides delocalized unpaired electrons and modulates a low coordination environment of adjacent metal sites, which is conducive to strengthen CO_2_ adsorption and finally accelerate the electrocatalytic kinetics for CO_2_RR/CO_2_ER processes.^[^ ^38^ ^]^ Moreover, oxygen vacancies can effectively tune the electronic states of multi‐metal oxides to enhance the conductivity, resulting in lowering the discharge‐charge polarization of Li‐CO_2_ batteries. The existence of these unpaired electrons trapped in the oxygen vacancies can be substantiated by the electron paramagnetic resonance (EPR) technique. As shown in Figure 2g and Figure S3 (Supporting Information), it can be evidently observed that Re‐NCM‐H3 possesses a stronger paramagnetic absorption peak than that of Re‐NCM at g value of 2.004, demonstrating more oxygen vacancies in Re‐NCM‐H3 catalyst.^[^ ^40^ ^]^

**Figure 3:**
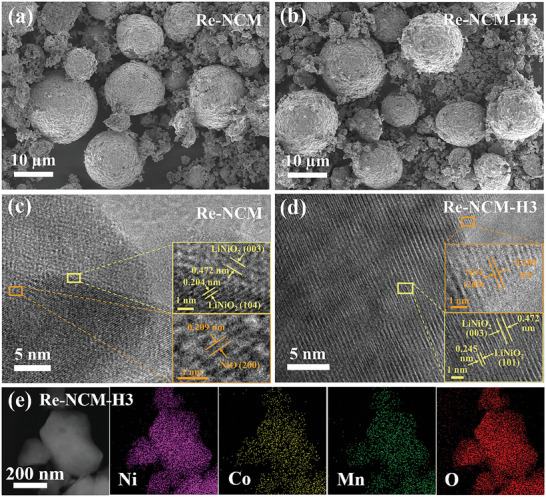
SEM images of a) Re‐NCM and b) Re‐NCM‐H3. High‐resolution TEM images of c) Re‐NCM and d) Re‐NCM‐H3. e) The corresponding EDX elemental mapping images of Re‐NCM‐H3.

The morphology changes and microstructure of the Re‐NCM material during H_2_‐annealing reduction were investigated by field‐emission scanning electron microscopy (FE‐SEM) and high‐resolution transmission electron microscopy (HR‐TEM), as presented in **Figure** **3** and S4–S8 (Supporting Information). The Re‐NCM sample consists of irregular spherical secondary particles and tiny primary particles with diameter ≈200–500 nm. After annealing at 300 °C, for the Re‐NCM‐H3 sample, the oxygen‐deficient feature scarcely changes the spherical morphology of secondary particles. However, the primary particles of Re‐NCM‐H3 are smaller size and rougher surface covered with cracks than those of the Re‐NCM sample, as seen in Figure 3b and Figure S4 (Supporting Information). This achieved smaller particle size after reduction not only guarantees a higher specific surface area, but also contributes to larger exposure of catalytic sites. The Brunauer–Emmett–Teller (BET) surface area of Re‐NCM‐H3 is 23.26 m^2^ g^−1^, which is higher than that of Re‐NCM (18.73 m^2^ g^−1^) (Figure S5, Figure 2). Furthermore, the SEM images and BET area results of Re‐NCM‐H4 and Re‐NCM‐H5 samples are provided in Figures S6 and S7 (Supporting Information). When the H_2_‐annealing temperature increases to above 400 °C, the morphological features of Re‐NCM‐H4 and Re‐NCM‐H5 samples have changed significantly along with their phase transformation obtained from the corresponding XRD analysis. This result has further proved that the annealing temperature of 300 °C is optimal. The HR‐TEM images of both Re‐NCM and Re‐NCM‐H3 (shown in Figure 3c,d) display clear lattice fringes of 0.204, 0.245, and 0.472 nm, corresponding to the (104), (101), and (003) planes of the layered LiNiO_2_, respectively. Meanwhile, a small region of these two samples has appeared lattice spacing of 0.209 nm indexed to the (200) plane of NiO, resulting from the Li^+^ deficiency and migration of Ni^2+^ into the Li layer during cycling. However, compared to Re‐NCM, the lattice fringes of NiO in the Re‐NCM‐H3 sample are blurry (depicted in Figure 3c,d), which can be ascribed to the reduced Ni^2+^ content and decreased crystallinity caused by the generation of oxygen vacancies. This is in accordance with the XRD and XPS results. Besides, the energy dispersive X‐ray spectroscopy (EDX) element mappings of Re‐NCM and Re‐NCM‐H3 samples are presented in Figure 3e and Figure S8 (Supporting Information). It can be seen that the elements of Ni, Co, Mn, and O are uniformly distributed on the surface of both Re‐NCM and Re‐NCM‐H3 samples. Based on the results obtained from XRD, XPS, EPR, FE‐SEM, and HR‐TEM, it can be speculated that H_2_‐annealing treatment at optimal temperature is a very useful strategy to generate the oxygen defects in the layered multi‐metal oxide Re‐NCM from spent LIBs, which has capability of moderating the catalytic activity for rechargeable Li‐CO_2_ batteries.

### 2.2 Electrochemical Performance of Li‐CO_2_ Battery Based on Re‐NCM‐H3 Catalyst

**Figure 4:**
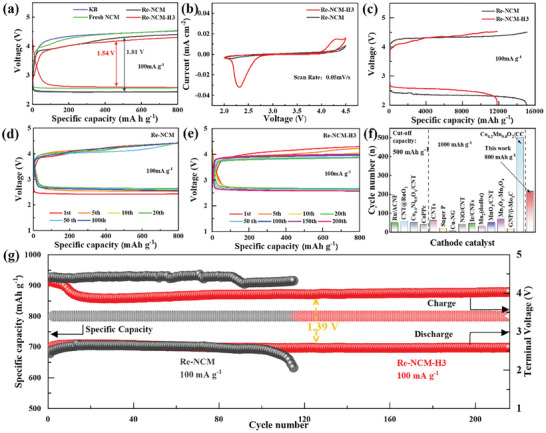
a) First discharge–charge profiles of Re‐NCM, Re‐NCM‐H3, KB and Fresh NCM based Li‐CO_2_ batteries. b) CV curves for Re‐NCM and Re‐NCM‐H3 at a scan rate of 0.05 mV s^−1^ within a voltage window of 2.0–4.5 V. c) Full discharge–charge profiles of the Li‐CO_2_ batteries with Re‐NCM or Re‐NCM‐H3 catalysts at a current density of 100 mA g^−1^. Cycling performance of Li‐CO_2_ batteries based on d) Re‐NCM and e) Re‐NCM‐H3 cathode at 100 mA g^−1^ with a limited specific capacity of 800 mAh g^−1^. f) Comparison of the cycling performance between Re‐NCM‐H3 and prior reported cathodic catalysts. g) Terminal discharge/charge voltages of Li‐CO_2_ batteries based on Re‐NCM and Re‐NCM‐H3 catalysts at 100 mA g^−1^ with a limited specific capacity of 800 mAh g^−1^.

In order to exclude the interference of Li^+^ insertion/extraction into the host structure of Re‐NCM and Re‐NCM‐H3 catalysts during discharge/charge cycles, first we assessed the discharge/charge behaviors of the electrodes with Re‐NCM and Re‐NCM‐H3 catalysts in the closed Li‐ion battery with ether‐based electrolyte, respectively. As shown in Figure S9 (Supporting Information), both of the closed Li‐ion batteries with the Re‐NCM and Re‐NCM‐H3 electrodes have hardly delivered any capacity no matter during discharging or during charging. Moreover, both of their charging voltage have rapidly increased to 4.8 V, which indicates that these as‐prepared Re‐NCM and Re‐NCM‐H3 catalysts with the layered structure themselves are durable and robust in the potential window from 2.0 to 4.5 V. Subsequently, to explore the influence of oxygen vacancies on the catalytic performance of the as‐prepared multi‐metal oxides Li‐Ni‐Co‐Mn recycled from spent LIBs, this research compared the electrochemical behaviors of rechargeable Li‐CO_2_ cells with different cathodic catalysts. **Figure** **4**a displays the initial discharge/charge profiles of Li‐CO_2_ batteries with the Re‐NCM, Re‐NCM‐H3, commercial ketjen black (KB) and fresh NCM811 cathodes at a cut‐off capacity of 800 mAh g^−1^ with a current density of 100 mA g^−1^. The Li‐CO_2_ cell with the fresh NCM catalyst operates with a relatively stable discharge platform of 2.55 V, and a much lower overpotential of 1.86 V than that of KB electrode (2.01 V). As expected, the discharge–charge voltage gap of battery based on Re‐NCM catalyst is mitigated to 1.81 V and the charge voltage reduces to 4.23 V. When it comes to the Li‐CO_2_ cell with Re‐NCM‐H3 catalyst, the discharge platform elevates to 2.60 V and the overpotential gap also remarkably reduces to 1.54 V. And the corresponding round‐trip energy efficiency can be measured up to 62.8%. It needs to be emphasized that the overpotentials were calculated from the charge–discharge voltage at 400 mAh g^−1^. Similar results can also be obtained in the cyclic voltammetry (CV) curves shown in Figure 4b. Obviously, the Re‐NCM‐H3 cathode delivers the higher onset potential for CO_2_RR (≈2.75 V), the lower onset potential for CO_2_ER (near 3.90 V) and the much larger peak current than those of Re‐NCM. This result has demonstrated that the incorporation of moderate oxygen vacancies not only boosts the kinetics of CO_2_ reduction, but also improves the catalytic activity for CO_2_ER during charge. Figure 4c depicts the full discharge/charge profiles of Li‐CO_2_ batteries with Re‐NCM and Re‐NCM‐H3 catalysts with the terminal voltage of 2.0 V and at a current density of 100 mA g^−1^, respectively. Although the Li‐CO_2_ cell based on Re‐NCM‐H3 catalyst exhibits smaller specific capacity of 11 808.9 mAh g^−1^, it can operate with a higher discharge platform (near 2.6 V) compared to that of the Re‐NCM (below 2.5 V), which further proves that the generated oxygen vacancies after H_2_‐annealing treatment can indeed accelerate the kinetics of CO_2_ adsorption and reduction reaction. It should be noted that the specific capacity is calculated based on the mass of active catalysts. In addition, the cycling performance of Li‐CO_2_ batteries with four different catalysts (including Re‐NCM, Re‐NCM‐H3, commercial KB and fresh NCM811, respectively) made a comparison at a current density of 100 mA g^−1^ with the cut‐off specific capacity of 800 mAh g^−1^. As shown in Figure 4d,e,g and Figure S10 (Supporting Information), the Li‐CO_2_ cell with Re‐NCM‐H3 catalyst possesses superior cyclability in contrast with those cells based on Re‐NCM, KB, fresh NCM catalysts. And the discharge and charge terminal voltages of Re‐NCM‐H3 based cell can still maintain at 2.57 and 4.02 V, respectively, after 216 cycles. However, the terminal discharge voltage of those Li‐CO_2_ batteries with KB, Re‐NCM has decayed below 2 V after 75 and 115 cycles, respectively. Moreover, the overpotential of Re‐NCM‐H3 based cell remains much lower than those of KB (2.27 V), fresh NCM (1.89 V) and Re‐NCM (1.42 V), verifying the outstanding catalytic activity of Re‐NCM‐H3 bearing with oxygen vacancies for efficiently boosting the CO_2_RR and CO_2_ER kinetics. Furthermore, as show in Figure 4f and Table S3 (Supporting Information), the cycling stability of Li‐CO_2_ battery with Re‐NCM‐H3 catalyst is much better than that of the batteries with present reported cathodic catalysts. Besides, the cycling performance of Li‐CO_2_ cells based on Re‐NCM‐H4 and Re‐NCM‐H5 are evaluated in Figure S11 (Supporting Information).

**Figure 5:**
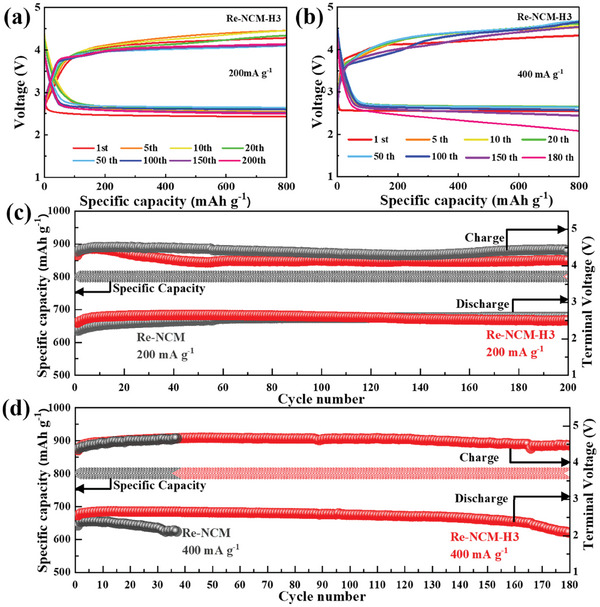
Rate cycling capability of the assembled Li‐CO_2_ batteries with Re‐NCM‐H3 catalyst at different current densities: a) 200 mA g^−1^, b) 400 mA g^−1^ with a limited specific capacity of 800 mAh g^−1^. The terminal discharge/charge voltages of Li‐CO_2_ batteries based on Re‐NCM and Re‐NCM‐H3 catalysts at c) 200 mA g^−1^ and d) 400 mA g^−1^ with a limited specific capacity of 800 mAh g^−1^.

The rate capability of Li‐CO_2_ batteries with the Re‐NCM and Re‐NCM‐H3 catalysts reused from spent LIBs is further investigated in **Figure** **5** and Figure S12 (Supporting Information). In stark contrast to the Re‐NCM electrodes, the Li‐CO_2_ cell with Re‐NCM‐H3 catalyst has delivered lower discharge/charge overpotentials and longer cycle life at the same current density. Specifically, the overpotential of Re‐NCM‐H3 based cell is 1.64 V at 200 mA g^−1^ with durability up to 200 cycles, which is distinctly smaller than the value of 1.85 V for Re‐NCM based battery. Even at an extremely high current density of 400 mA g^−1^, the initial voltage gap of Re‐NCM‐H3 based battery is as small as 1.78 V, whereas the cell with Re‐NCM has presented an overpotential gap of 2.08 V. Here the overpotential gaps are calculated by the terminal discharge/charge potentials. Moreover, as shown in Figure 5d, the Re‐NCM‐H3 based cell can stably operate over 165 cycles at 400 mA g^−1^, while the battery with the Re‐NCM catalyst maintains just 35 cycles. These results have provided further evidence that appropriate oxygen vacancies can enhance the rate capability and cycling durability for Li‐CO_2_ batteries with Re‐NCM‐H3 catalyst. This is due to the accelerated electron transfer aroused by the appearance of oxygen vacancies, which has been affirmed by fitted electrochemical impedance spectroscopy (EIS) results in Figure S13 (Supporting Information). Comparing with the corresponding fitting results listed in Table S4 (Supporting Information), both the ohmic resistance (R_1_) (13.74 Ω) and charge transfer resistance (R_2_) (303.1 Ω) on the Re‐NCM‐H3 electrode are significantly decreased than those of Re‐NCM (R_1_ = 33.9 Ω, R_2_ = 435.3 Ω). As a consequence, the oxygen vacancy exerts a critical role in promoting the charge transfer kinetic on the surface of Re‐NCM‐H3 catalyst.

### 2.3 Ex Situ Characterization of the Re‐NCM‐H3 Catalyst‐Based Li‐CO_2_ Batteries

**Figure 6:**
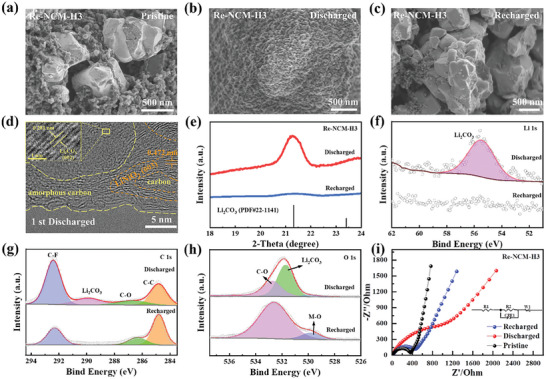
Morphology and structural evolution of Re‐NCM‐H3 electrode at different stages. SEM images of a) pristine, b) full discharged and c) full recharged Re‐NCM‐H3 cathode. d) High‐resolution TEM images of full discharged carbon‐free Re‐NCM‐H3 cathode. e) XRD patterns, f) Li 1s, g) C 1s, h) O 1s XPS spectra of discharged and recharged Re‐NCM‐H3 cathode. i) EIS spectra of pristine, discharged and recharged Re‐NCM‐H3 cathode. The inset displays the corresponding equivalent circuit.

To further elucidate the influence of oxygen vacancies on the electrochemical mechanisms of Li‐CO_2_ batteries during the discharge/charge process, the deposition morphology and composition evolution of discharge products on different cathodes were detected by ex situ SEM and TEM. As shown in **Figure** **6**b, many uniform granular‐like discharge products with a size range of 50–100 nm were formed on the surface of the Re‐NCM‐H3 cathode after discharged to 2.3 V. After full recharge, these particles entirely disappeared and the Re‐NCM‐H3 electrode has returned to its pristine morphology (Figure 6a), demonstrating the desirable rechargeability of discharge products. Compared with the Re‐NCM based electrode, the agglomerated discharge products composed of flake layers can be clearly observed to grow throughout the Re‐NCM electrode surface upon discharge (Figure S14, Supporting Information), but there exist a few minor undecomposed products in the subsequent charge process. A typical region on the carbon‐free Re‐NCM‐H3 electrode after discharged to 2.3 V has been detected in the HR‐TEM image as presented in Figure 6d and Figure S15 (Supporting Information). The measured lattice spacing is 0.281 nm, which corresponds to the (002) crystal plane of Li_2_CO_3_ (PDF 22‐1141). Apart from the crystalline Li_2_CO_3_, the (003) plane of LiNiO_2_ and amorphous carbon can be also found. This result offers a proof for the formation of Li_2_CO_3_ and carbon during the discharge process. The XRD patterns in Figure 6e and Figure S16 (Supporting Information) have demonstrated that even though the morphologies of the discharge products in Li‐CO_2_ cells with Re‐NCM‐H3 or Re‐NCM catalyst are totally different, the diffraction peaks of Li_2_CO_3_ crystallites were detected both on the Re‐NCM‐H3 and Re‐NCM electrode after discharge. And the distinct Li_2_CO_3_ diffraction peak at 21.3 ° almost disappeared on the Re‐NCM‐H3 cathode, whereas the Re‐NCM electrode cannot completely recovered its pristine composition. These results have fully reflected that the Re‐NCM‐H3 material has superior capability of catalyzing the formation and decomposition of Li_2_CO_3_. To further confirm the satisfying reversibility of the Li‐CO_2_ battery catalyzed by Re‐NCM‐H3, the XPS spectra of Li 1s, C 1s, and O 1s were here recorded respectively for the discharged and recharged Re‐NCM‐H3 cathode (Figure 6f,g,h). Evidently, the Li 1s peak at 55.44 eV which caused by the generated Li_2_CO_3_ can be detected after discharge, which was completely removed after full recharging (Figure 6f). In addition, the C 1s spectra can be divided into three peaks located at 284.8, 286.6, and 292.4 eV attributing to the C─C, C─O, and C─F bonds, respectively.^[^ ^41^ ^]^ After the discharge process, the characteristic peak of Li_2_CO_3_ at 289.9 eV emerged, which was removed completely at the end of recharging.^[^ ^41^ ^]^ These results can be corroborated by the O 1s XPS analysis. Moreover, the Ni 2p, Co 2p, and Mn 2p XPS spectra of the Re‐NCM‐H3 electrode at different stages are displayed in Figure S17 (Supporting Information), to investigate the surface state changes of multi‐metal Li‐Ni‐Co‐Mn oxide during discharge and charge. It can be found that the valence states of Ni and Co remain almost unchanged during the discharge and recharge process. And the signal peaks of Mn 2p cannot be detected after discharge probably because of the coverage of Li_2_CO_3_ and/or carbonaceous species on the Re‐NCM‐H3 electrode, which emerged again after the decomposition of discharging products. Besides, the EIS plots and equivalent circuits of the Li‐CO_2_ batteries with Re‐NCM‐H3 electrode at different stages are shown in Figure 6i. The corresponding EIS fitting results are listed in Table S5 (Supporting Information). For the fully discharged Re‐NCM‐H3 electrode, an enlarged charge transfer impedance can be obtained due to accumulation of the insulating Li_2_CO_3_. After the subsequent recharging process, the ability of charge transfer can be largely resumed to that of the pristine electrode, indicating the efficient decomposition of discharge products during recharge.

### 2.4 Theoretical Understanding on the Origin of Excellent Catalytic Activity on CO_2_RR/CO_2_ER

**Figure 7:**
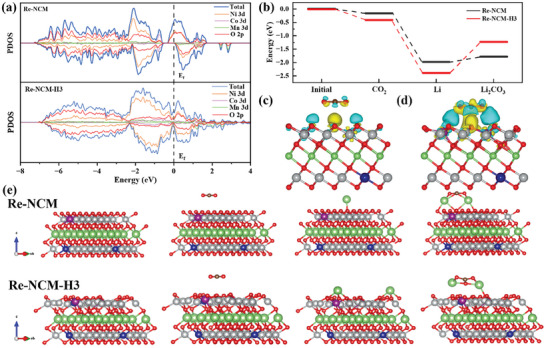
a) Comparison of PDOS of Re‐NCM and Re‐NCM‐H3 catalyst. b) Calculated energy profiles of CO_2_, Li, Li_2_CO_3_ on the surface of Re‐NCM and Re‐NCM‐H3. Charge density difference of c) Re‐NCM d) Re‐NCM‐H3 with surface absorbed CO_2_ molecular (yellow for Δρ > 0 and blue for Δρ < 0). e) Configurations of Re‐NCM, Re‐NCM‐H3 and the corresponding CO_2_, Li and Li_2_CO_3_ absorption sites. Green balls represent Li atoms, gray balls represent Ni atoms, blue balls represent Co atoms, purple balls represent Mn atoms, red balls represent O atoms.

In order to deeply comprehend the substantial reason for the enhancement of the electrochemical behaviors of Li‐CO_2_ battery with Li‐Ni‐Co‐Mn oxide catalyst caused by more oxygen vacancies, a series of spin‐polarized DFT calculations were executed to explore the influence of oxygen vacancy on the electronic structure of Re‐NCM and catalytic activity of CO_2_RR/CO_2_ER. As shown in **Figure** **7**a, compared with the partial density of states (PDOS) of the Re‐NCM, Re‐NCM‐H3 not only fails to exist a gap at the Fermi level (E_f_) but also appears extra electronic states close to the E_f_, indicating good electronic conductivity caused by the introduction of oxygen vacancies.^[^ ^42^, ^43^ ^]^ Figure 7b depicts the adsorption energies of Li, CO_2_ and Li_2_CO_3_ on the Re‐NCM and Re‐NCM‐H3, respectively. The adsorption energy of CO_2_ on Re‐NCM and Re‐NCM‐H3 are −0.16 and −0.41 eV, respectively, confirming that CO_2_ prefers to be adsorbed on the Re‐NCM‐H3. Therefore, the oxygen vacancies on Re‐NCM‐H3 contribute to enhance the CO_2_RR kinetics. Additionally, the Re‐NCM‐H3 exhibits weaker Li_2_CO_3_ adsorption capacity compared to the Re‐NCM, which suggests that Li_2_CO_3_ on the Re‐NCM‐H3 can be decomposed easier than that of Re‐NCM during the CO_2_ER process.^[^ ^44^ ^]^ Aiming to explore the essential reason, the differential charge density Δρ of CO_2_ adsorbed on the surface of Re‐NCM and Re‐NCM‐H3 are calculated (Figure 7c,d). On the surface of Re‐NCM‐H3, a net loss of electronic charge and electronic gathering can be observed obviously between Re‐NCM‐H3 and CO_2_ molecular (blue area and yellow area). This further confirms that the introduce of oxygen vacancy makes the charge distribution on the Re‐NCM‐H3 surface inhomogeneous, which facilitates the charge redistribution on the CO_2_ molecular during the adsorption.^[^ ^45^ ^]^ In other words, the introduce of oxygen vacancy intensify the charge transfer between CO_2_ molecular and Re‐NCM‐H3, and then enhance the adsorption ability of Re‐NCM‐H3 onto CO_2_ molecular. In order to visualize the adsorption process, the modeling and adsorption configurations of Re‐NCM and Re‐NCM‐H3 are shown in the Figure 7e. Based on the theoretical calculations above, the exist of oxygen vacancy accelerates the charge transfer, leading to high catalytic performance of Re‐NCM‐H3 in Li‐CO_2_ batteries.

## 3 Conclusion

Compared with conventional recycling procedures, direct reuse of metal resources from spent ternary LIBs like in this work can not only prevent intricate processes and secondary environmental pollution, but also provide huge economic profits. This paper first successfully presented a strategy for improving the catalytic activity of recovered Li‐Ni‐Co‐Mn oxides from spent NCM cathode on CO_2_RR and CO_2_ER by engineering oxygen vacancy. The oxygen vacancy‐bearing Li‐Ni‐Co‐Mn multi‐metal oxide (Re‐NCM‐H3) catalyst was obtained, without changing the *α*‐NaFeO_2_‐type structure, by annealing the NiCoMn precursor (Re‐NCM) in H_2_ at the optimum temperature of 300 °C. DFT calculations coupled with experimental results have testified that the creation of moderate oxygen vacancies can effectively tailor the electronic structure of Re‐NCM, which consolidates both the adsorption behavior of carbonaceous species and electronic conductivity of Re‐NCM‐H3, and thus improving the catalytic capability of Re‐NCM‐H3 for rechargeable Li‐CO_2_ batteries. Profiting from the synergistic implication of multi‐metal active sites and oxygen vacancies, the Li‐CO_2_ battery with Re‐NCM‐H3 catalyst has delivered a lower overpotential of 1.54 V, and prolonged cycling stability over 216 cycles at 100 mA g^−1^ and 165 cycles at 400 mA g^−1^ with a cut‐off specific capacity of 800 mAh g^−1^. Furthermore, various ex situ characterizations, such as SEM, TEM, XPS et al., have been carried out to substantiate the reversible formation/decomposition of discharged products (Li_2_CO_3_ and carbon) in the assembled Li‐CO_2_ battery with Re‐NCM‐H3 catalyst. Our findings provide a sustainable method to turn “waste” LIBs to wealth and give useful guidance for designing low‐cost and high‐efficiency multi‐metal oxide catalysts based on defect engineering in Li‐CO_2_ batteries.

## 4 Experimental Section

### Preparations of Materials—Preparation of the NiCoMn Precursor from a Spent NCM811 Cathode

The retired 18650‐type LIBs with a ternary LiNi_0.8_Co_0.1_Mn_0.1_O_2_ cathode were collected from Suzhou YouLion Battery Inc., Suzhou, China. First, the spent LIBs were totally discharged, and then manually dismantled to separate the cathode scraps, anode and steel cases. Then, the obtained cathode (the active material NCM811 on Al foil current collector) was cut into tiny pieces using a scissors for further alkali leaching treatment. A solution of 1 m NaOH was used to remove aluminum, leading to separation of cathode active material NCM811 and Al foil. After filtrating and washing several times, the active material powder was calcinated in air at 500 °C for 3 h to remove conductive carbon black and binder, thus obtaining the NiCoMn precursor (donoted as Re‐NCM).

### Preparations of Materials—Preparation of the Oxygen Vacancy‐Bearing Li‐Ni‐Co‐Mn Multi‐Metal Oxide Catalyst Through the H_2_‐Annealing Treatment

The H_2_ annealing treatment was conducted in a tube furnace. The obtained NiCoMn precursor was placed in a crucible at the center of the tube, and 5% H_2_ in Ar was imported to the tube at a constant flow rate of 60 mL min^−1^. The samples were annealed at 300, 400, and 500 °C, respectively, under the 5% H_2_/Ar flow. The temperature rised at a rate of 5 °C min^−1^, and kept at the given temperature for 3 h. After annealing, the three samples were cooled down to the room temperature, which were denoted as Re‐NCM‐H3, Re‐NCM‐H4, Re‐NCM‐H5, respectively.

### Material Characterizations

X‐ray diffraction (XRD, SmartLabSE, Rigaku Corporation, Japan; Cu K*α* radiation) was used to characterize the crystal structure. Scanning Electron Microscope (SEM, Hitachi SU8100, Japan) and Transmission Electron Microscope (TEM, JEOL JEM‐F200, Japan) were adopted to investigate the surface morphology and microstructure of the as‐prepared catalysts and discharge products. X‐ray photoelectron spectroscopy (XPS, Thermo Fisher Nexsa, UK) was utilized to analyze the chemical composition and valence states of as‐prepared Re‐NCM and Re‐NCM‐H3 catalysts. Electron paramagnetic resonance (EPR) signals were detected by Bruker EMX PLUS to investigate the oxygen vacancies. Inductively coupled plasma optical emission spectrometer (ICP‐OES) using Agilent 5110, USA was used to measure the chemical compositions of Re‐NCM and Re‐NCM‐H3 catalysts. Particle size distribution histograms were detected by particle size distribution histogram.

### Battery Assembly and Electrochemical Tests

The fabrication of CO_2_ cathode was elaborated as follows. Active catalyst (Re‐NCM or Re‐NCM‐H3), Ketjen black (KB) and polyvinylidene fluoride (PVDF) binder with a mass ratio of 8:1:1 were intimately mixed and dispersed in the N‐methyl 2‐pyrrolidone (NMP) solvent. The slurry was evenly casted on the carbon paper substrate (12 mm diameter, Toray), and subsequently dried at 120 °C under vacuum for 12 h. The cathode loading was 0.15–0.20 mg cm^−2^. The testing CR2032‐type coin cells were assembled in an Ar‐filled glove box, in which both moisture and oxygen levels below 0.1 ppm. The cathode shell was machine‐drilled with seven small holes with a diameter of 2.0 mm as CO_2_ gas passages. Furthermore, a lithium foil (diameter: 14 mm and thickness: 0.6 mm) was used as the battery anode, and a glass fiber (GF/B, Whatman) was used as the separator. And 1 m lithium bis (trifluoromethyl sulphonyl) imide (LiTFSI) / tetraethylene glycol dimethyl ether (TEGDME) was employed as the aprotic electrolyte. The electrolyte amount in a cell was measured to 0.1 mL by a pipette.

Galvanostatic discharge–charge cycling tests were conducted with a LANHE battery testing system (CT3002A, Wuhan, China). Both cyclic voltammetry (CV) and electrochemical impedance test (EIS) were performed using a CHI 760E electrochemical workstation (Chenhua Corp., Shanghai, China). The EIS frequency ranges from 100 kHz to 0.01 Hz. All the battery tests were conducted at a constant temperature of 25 °C and in a sealed chamber filled with high‐purity CO_2_.

### Theoretical Methods

Spin‐polarized DFT calculations using Vienna ab initio simulation package (VASP) were employed in order to investigate the binding among materials.^[^ ^46^, ^47^ ^]^ The generalized gradient approximation with Perdew–Burke–Ernzerhof (GGA‐PBE) exchange‐correlation functional was picked for the exchange‐correlation energy.^[^ ^48^ ^]^ The projector‐augmented‐wave (PAW) approach was adopted to analyze the pseudo‐potential.^[^ ^49^ ^]^ The computationally cost‐effective Grimme's D3 scheme method with Becke‐Jonson damping for the van der Waals (vdW) interactions was selected to properly represent the dispersion interaction between various intermediates and electrode material.^[^ ^50^ ^]^ The geometry optimization was carried out without any symmetry constraint, until the computed Hellmann–Feynman force on all the atoms were less than 0.05 eV Å^−1^. The energy criterion of 10^−4^ eV was used in the iterative solution of the Kohn–Sham equation. For the optimization and self‐consistent calculations of Re‐NCM and Re‐NCM‐H3 supercells, the Brillouin zone was sampled with 2×2×1 Monkhorst–Pack k‐point mesh. The electron density differences were plotted in VESTA software. In order to speed up the electronic convergence, a Gaussian smearing of 0.05 eV was employed. To eliminate the undesirable interaction between the slab and its period images, a vacuum layer of 15 Å was established along the vertical direction.

The adsorptions of CO_2_, Li and Li_2_CO_3_ molecules on Re‐NCM or Re‐NCM‐H3 surface were calculated respectively. Herein, the adsorption energy ΔE*_ads_* was calculated by the equation: ΔE*_ads_ =* E*_M/surface_* – E*_surface_* – E*_M_*, where E*_M/surface_*, E*_surface_* represent the total energy of catalyst with and without the molecule M adsorbed on surface respectively and E*_M_* is the energy molecule. Specifically, the more negative value of ΔE*_ads_* implies the stronger adsorption between molecule and catalyst surface.

## Conflict of Interest

The authors declare no conflict of interest.

## Supporting information

Supporting Information

## Data Availability

The data that support the findings of this study are available from the corresponding author upon reasonable request.
