# Supplementary material for: Improving the Rechargeable Li‐CO2 Battery Performances by Tailoring Oxygen Defects on Li‐Ni‐Co‐Mn Multi‐Metal Oxide Catalysts Recycled from Spent Ternary Lithium‐Ion Batteries
Source: Adv Sci (Weinh). 2024 May 17;11(28):2402892. doi: 10.1002/advs.202402892 (PMC11267390; doi:10.1002/advs.202402892)
Supplement: Supplementary file 1 — Supporting Information [file ADVS-11-2402892-s001.pdf]

ADVANCED SCIENCE
Open Access

Supporting Information

for *Adv. Sci.*, DOI 10.1002/advs.202402892

# Improving the Rechargeable Li-$CO_2$ Battery Performances by Tailoring Oxygen Defects on Li-Ni-Co-Mn Multi-Metal Oxide Catalysts Recycled from Spent Ternary Lithium-Ion Batteries

*Juan Wang, Ningning Feng*, Shuang Zhang, Yang Lin, Yapeng Zhang, Jing Du, Senlin Tian, Qun Zhao* and Gang Yang*

Supporting Information

# Improving the Rechargeable Li-$CO_2$ Battery Performances by Tailoring Oxygen Defects on Li-Ni-Co-Mn Multi-metal Oxide Catalysts Recycled from Spent Ternary Lithium-ion Batteries

*Juan Wang, NingNing Feng,* Shuang Zhang, Yang Lin, Yapeng Zhang, Jing Du, Senlin Tian, Qun Zhao* and Gang Yang*

J. Wang, S. Tian, Dr. Q. Zhao
Kunming University of Science and Technology, Kunming 650093, P.R. China
E-mail: zq441206@163.com

Dr. N. Feng, S. Zhang, Y. Lin, Y. Zhang, J. Du, G. Yang
Suzhou Key Laboratory of Functional Ceramic Materials Department, Changshu Institute of Technology, Suzhou 215500, P. R. China
E-mail: nningfeng@cslg.edu.cn

**Table S1** ICP-OES results of obtained samples.

| Sample | Li | Ni | Co | Mn |
|---|---|---|---|---|
| Re-NCM[a] | 28.245 | 235.6 | 27.67 | 25.135 |
| Re-NCM-H3[a] | 27.23 | 228.055 | 26.515 | 24.035 |
| Re-NCM[b] | 6.3047 | 52.5893 | 6.1763 | 5.6105 |
| Re-NCM-H3[b] | 7.0544 | 59.0816 | 6.8692 | 6.2267 |
| Re-NCM[c] | | $Li_{0.824}Ni_{0.812}Co_{0.095}Mn_{0.092}O_{1.661}$ | | |
| Re-NCM-H3[c] | | $Li_{0.822}Ni_{0.814}Co_{0.094}Mn_{0.092}O_{1.050}$ | | |

[a] concentration of each element in the prepared catalysts. (mg $L^{-1}$)

[b] Mass percentage of each element in the prepared catalysts.

[c] Molecular Formula of prepared catalysts.

The calculations of the molecular formulas were calculated by normalizing the three elements of Ni, Co and Mn.

**Table S2.** Concentration of oxygen species in Re-NCM and Re-NCM-H3

| Sample | $C_O$ (%) | $C_{Olattice}$ (%) | $C_{OV}$ (%) | $C_{OV}$/ $C_{Olattice}$ |
|---|---|---|---|---|
| Re-NCM | 53.2 | 29.97 | 20.03 | 2.25 |
| Re-NCM-H3 | 43.12 | 17.51 | 32.49 | 3.66 |

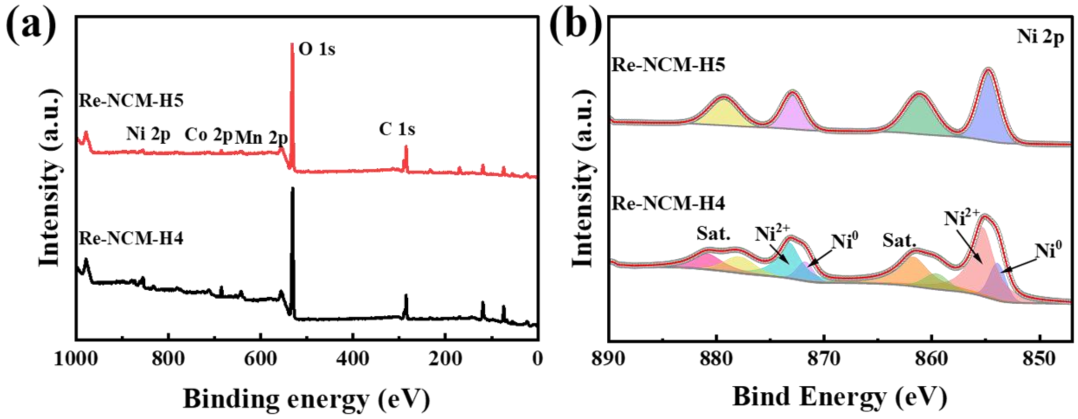


**Figure. S1.** a) Full XPS spectrum, b) Ni 2p spectra of Re-NCM-H4 or Re-NCM-H5.

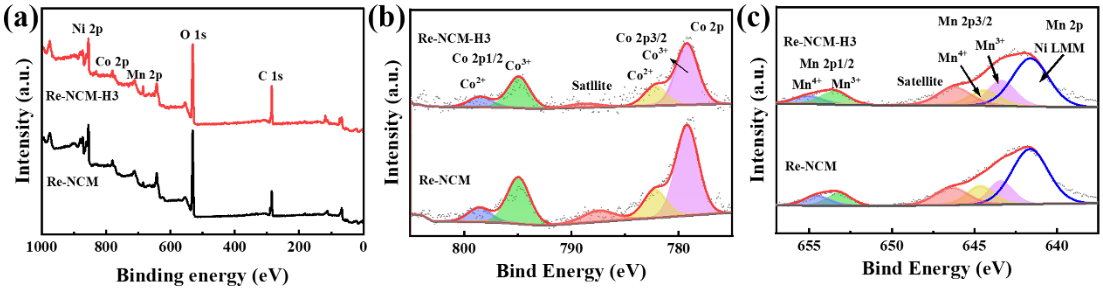


**Figure. S2.** a) Full XPS spectrum, b) Co 2p and c) Mn 2p XPS spectra of as-prepared Re-NCM or Re-NCM-H3 catalyst.

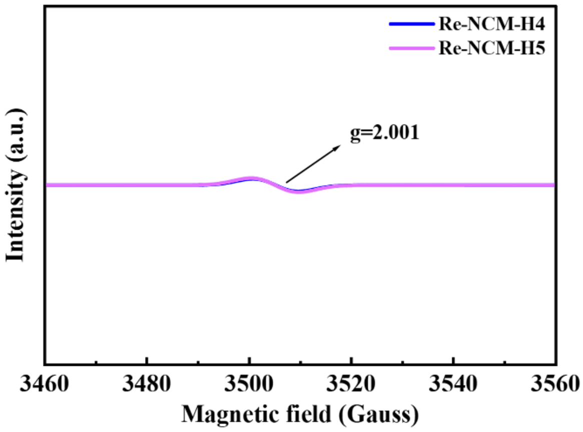


**Figure. S3** EPR spectra of Re-NCM-H4 and Re-NCM-H5.

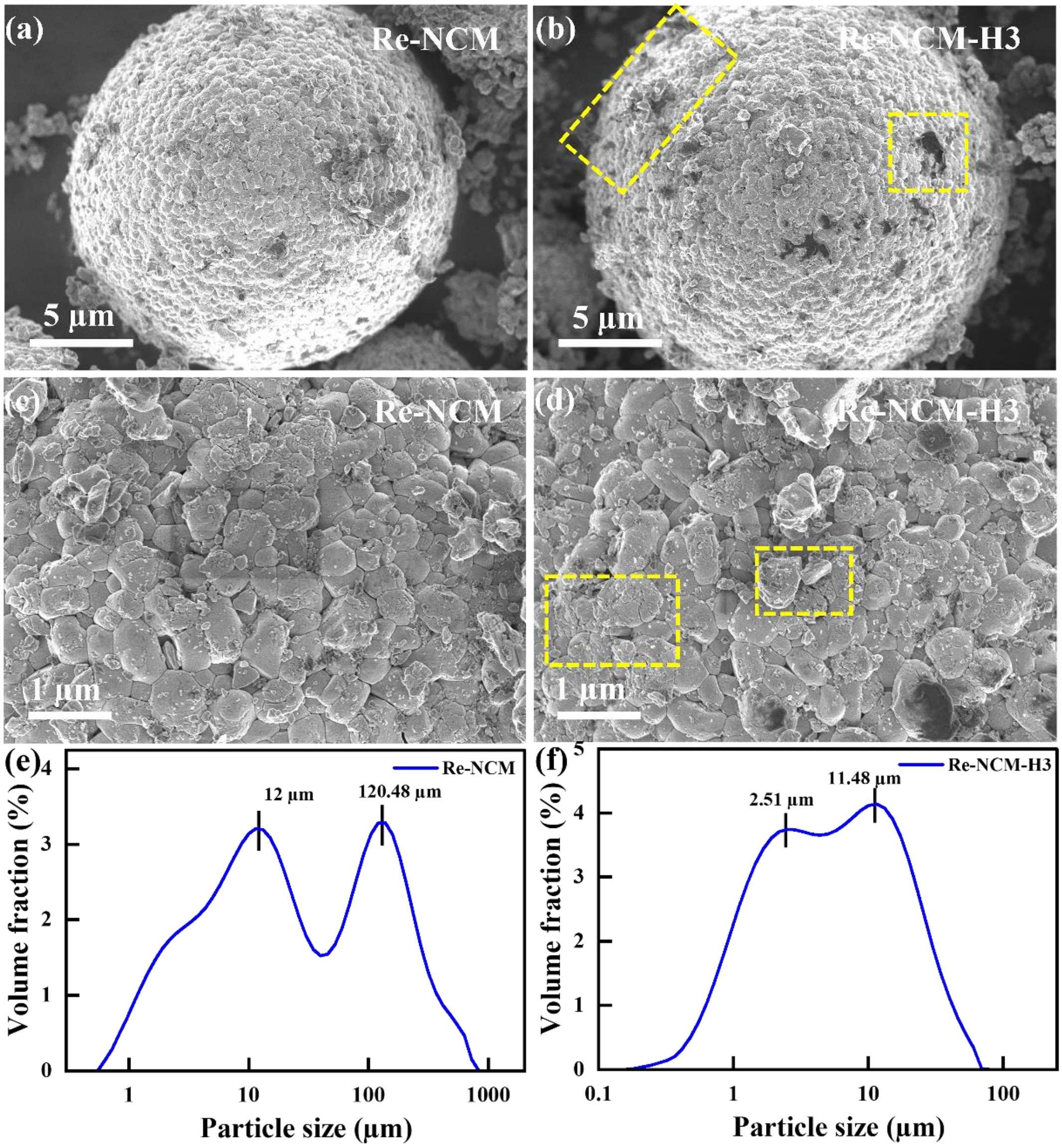


**Figure. S4.** a, b, c, d)SEM images e, f) Particle size distribution histogram of as-prepared Re-NCM and Re-NCM-H3.

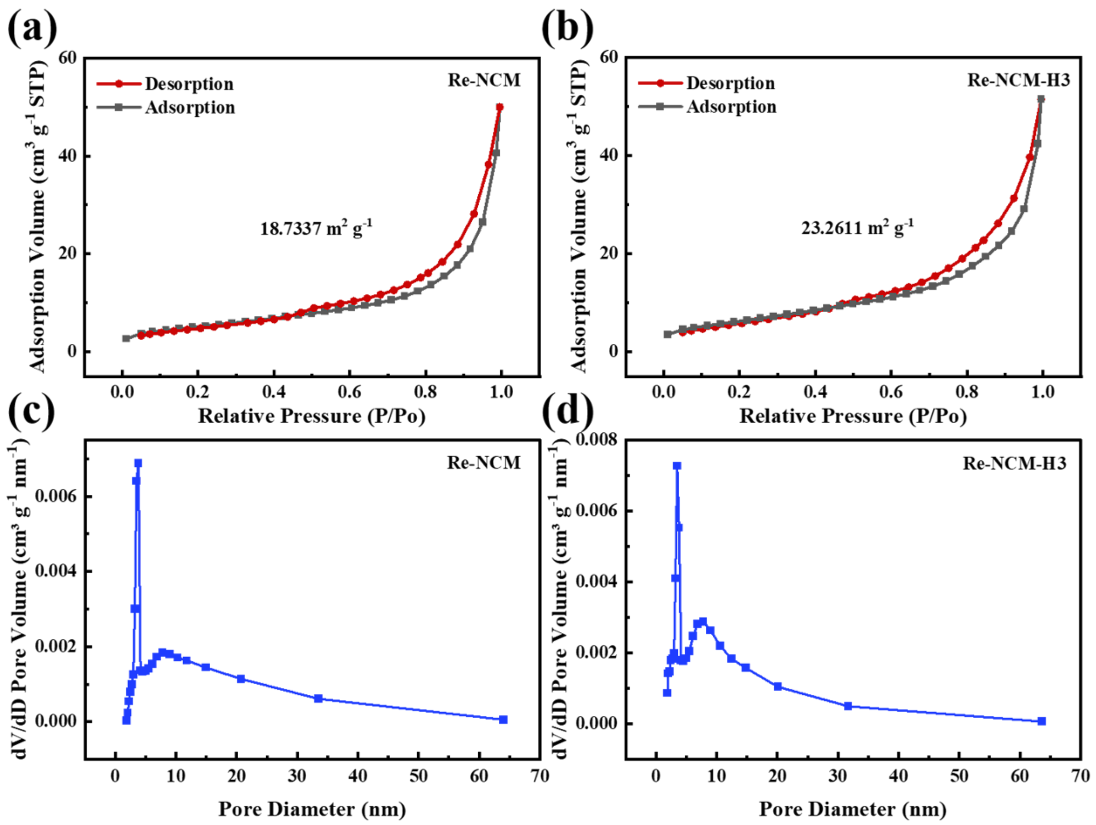


**Figure. S5.** a, b) $N_2$ sorption isotherms and c, d) the pore size distribution curves of Re-NCM and Re-NCM-H3.

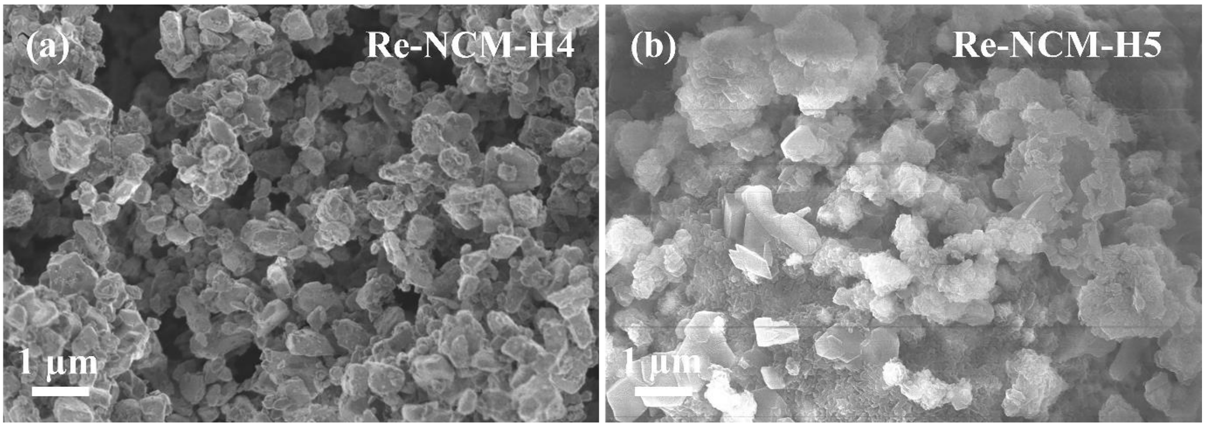


**Figure. S6.** SEM images of a) Re-NCM-H4 and b) Re-NCM-H5.

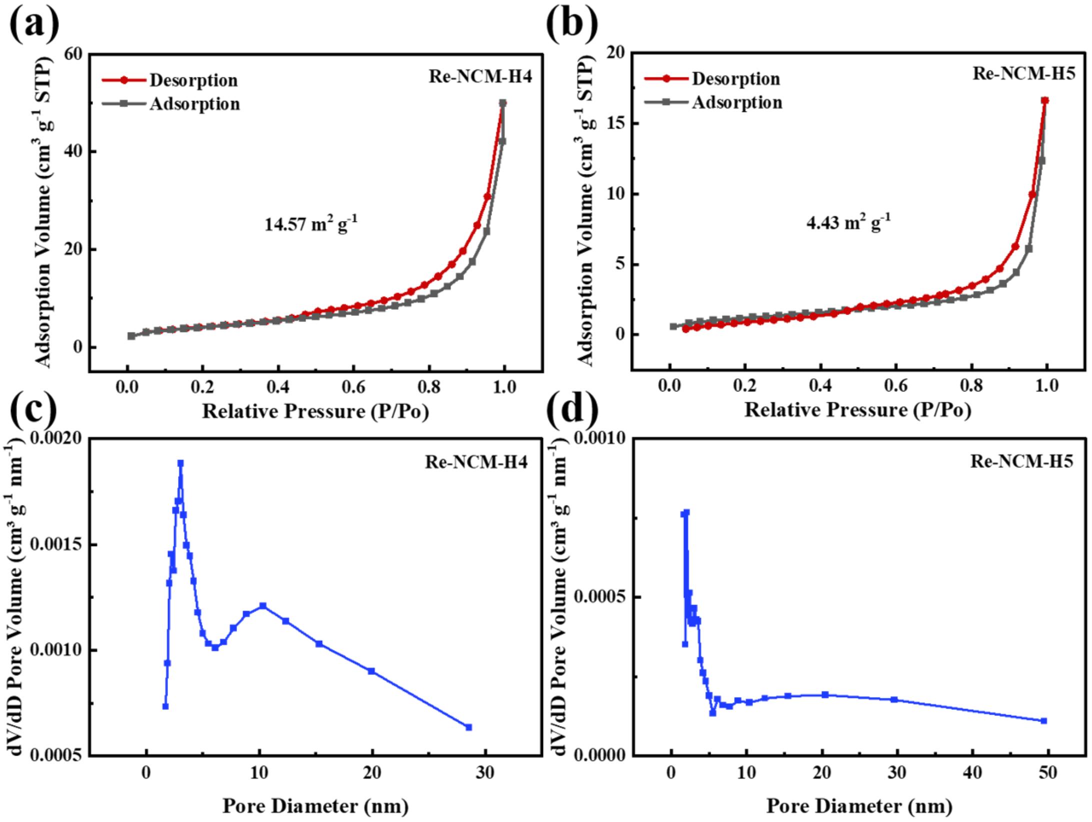


**Figure. S7.** a, b) $N_2$ sorption isotherms and c, d) the pore size distribution curves of Re-NCM-H4 and Re-NCM-H5.

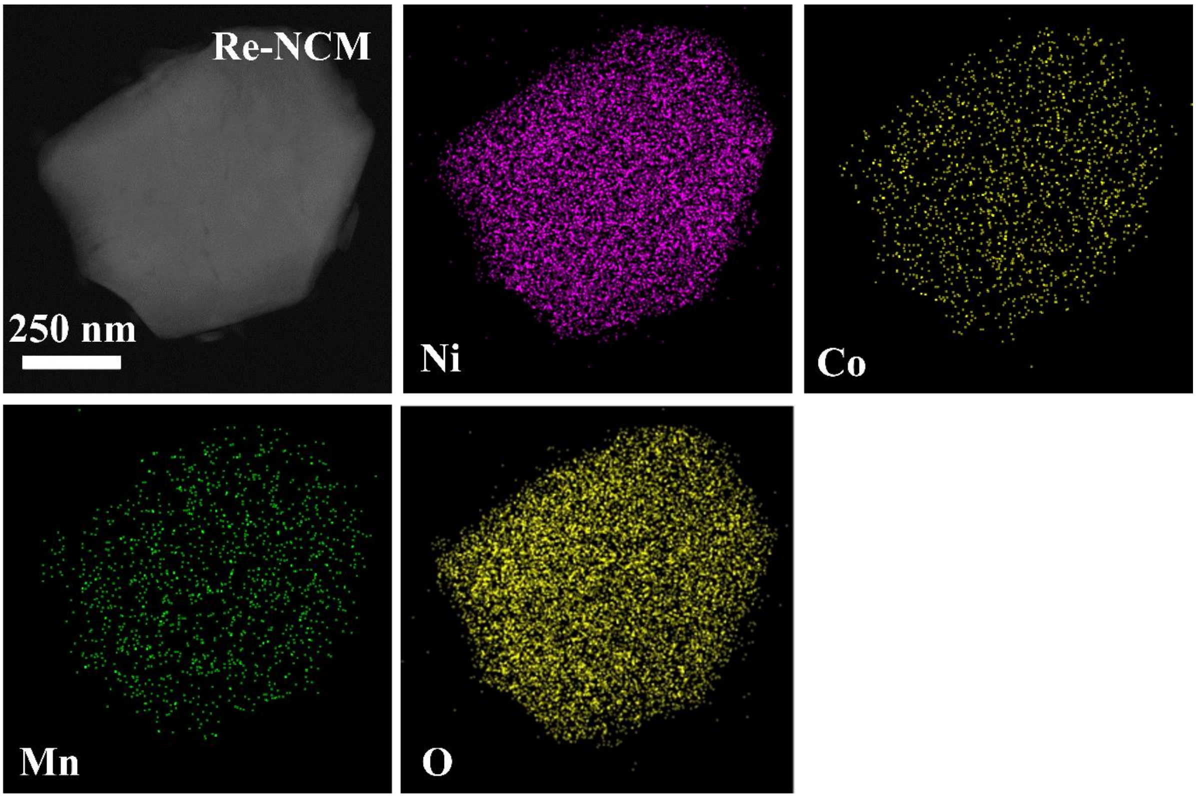


**Figure. S8.** TEM and corresponding EDX elemental mapping images of Re-NCM.

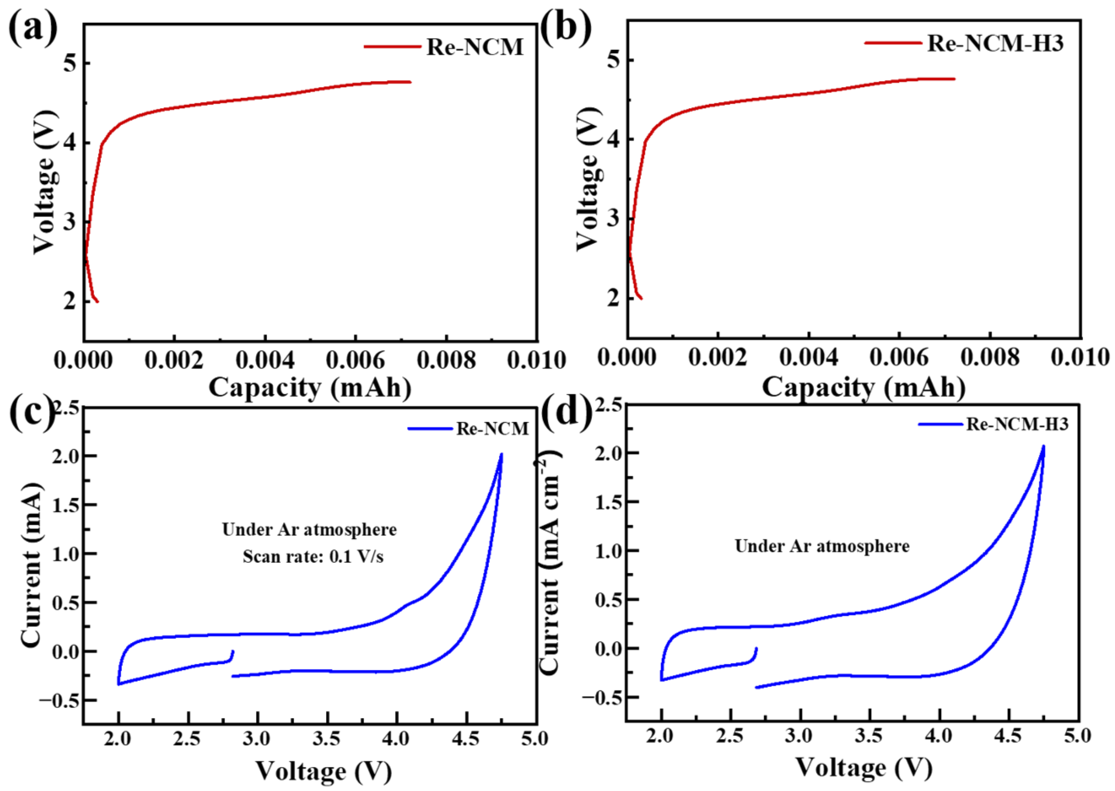


**Figure. S9.** a, b) First discharge/charge curves c, d) CV curves for the closed Li-ion batteries with Re-NCM and Re-NCM-H3 electrodes.

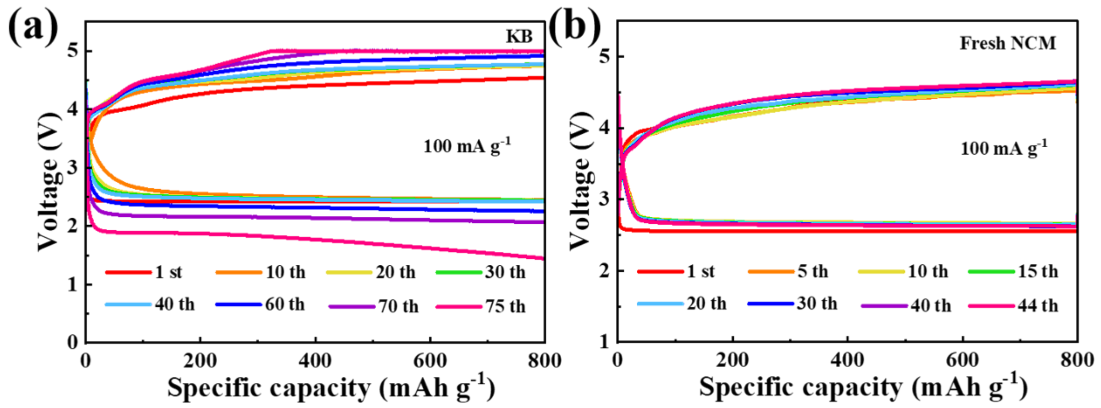


**Figure. S10.** Cycling performance of Li-$CO_2$ batteries based on a) KB and b) Fresh NCM cathodic catalyst at 100 mA g$^{-1}$ with a limited specific capacity of 800 mAh g$^{-1}$.

**Table S3.** Comparison and summary of recent literatures on cycle performance of Li-$CO_2$ batteries with different cathodes.

| Materials | Cycle numbers | Limited specific capacity (mAh $g^{-1}$) | References |
|---|---|---|---|
| **Re-NCM-H3** | **216** | **800** | **This work** |
| Ru/ACNF | 38 | 500 | *Ref.* S1 |
| CNT@$RuO_2$ | 55 | 500 | *Ref.* S2 |
| $Co_{0.1}Ni_{0.9}O_x$/CNT | 50 | 500 | *Ref.* S3 |
| CoPPc | 38 | 500 | *Ref.* S4 |
| CNTs | 60 | 1000 | *Ref.* S5 |
| Super P | 20 | 1000 | *Ref.* S6 |
| Cu-NG | 50 | 1000 | *Ref.* S7 |
| NiO/CNT | 42 | 1000 | *Ref.* S8 |
| Ir/CNFs | 45 | 1000 | *Ref.* S9 |
| $Mn_2$(dodbc) | 30 | 1000 | *Ref.* S10 |
| $MnO_2$/CNT | 50 | 1000 | *Ref.* S11 |
| $Mn_2O_3$-$Mn_3O_4$ | 69 | 1000 | *Ref.* S12 |
| GNP/β-$Mo_2C$ | 17 | 1000 | *Ref.* S13 |
| $Co_{0.2}Mn_{0.8}O_2$ | 500 | 1000 | *Ref.* S14 |

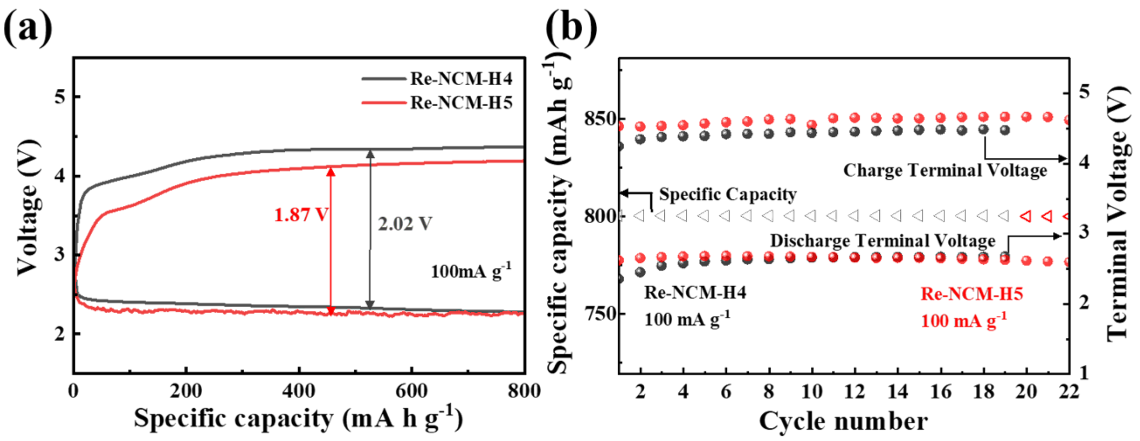


**Figure. S11.** a) First discharge-charge profiles and b) cycling performance of Li-$CO_2$ batteries based on Re-NCM-H4 and Re-NCM-H5 catalysts at 100 mA $g^{-1}$ with a limited specific capacity of 800 mAh $g^{-1}$.

The overpotentials were calculated from the charge-discharge voltage at 400 mAh $g^{-1}$.

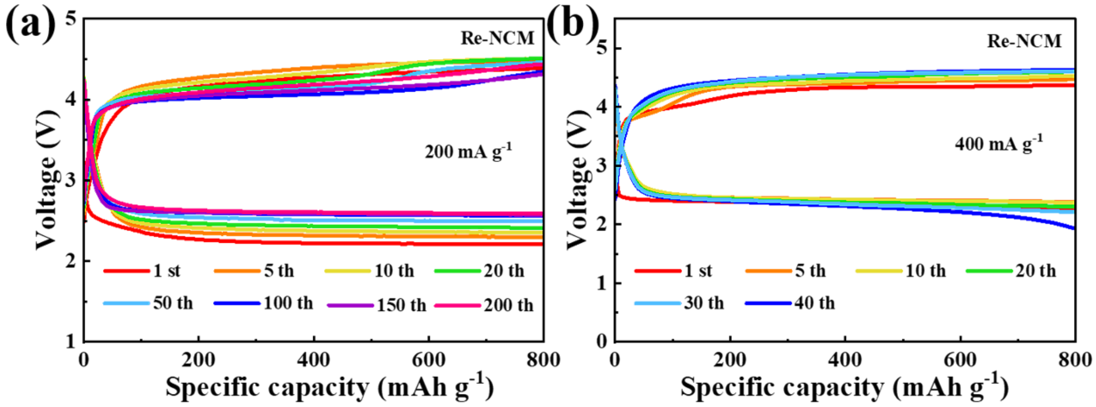


**Figure. S12.** Rate cycling capability of the assembled Li-$CO_2$ batteries with Re-NCM catalyst at different current densities: a) 200 mA $g^{-1}$, b) 400 mA $g^{-1}$ with a limited specific capacity of 800 mAh $g^{-1}$.

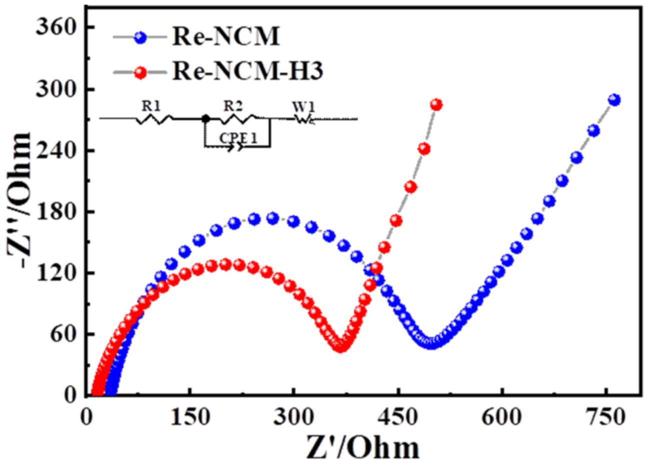


**Figure. S13.** EIS spectra of Li-$CO_2$ batteries with Re-NCM and Re-NCM-H3 cathodic catalysts, respectively.

**Table S4.** The Ohmic impedance and electron transfer resistance of Re-NCM and Re-NCM-H3 cathode

| Sample | $R_1$ (ohm) | $R_2$ (ohm) |
|---|---|---|
| Re-NCM | 33.9 | 435.3 |
| Re-NCM-H3 | 13.74 | 303.1 |

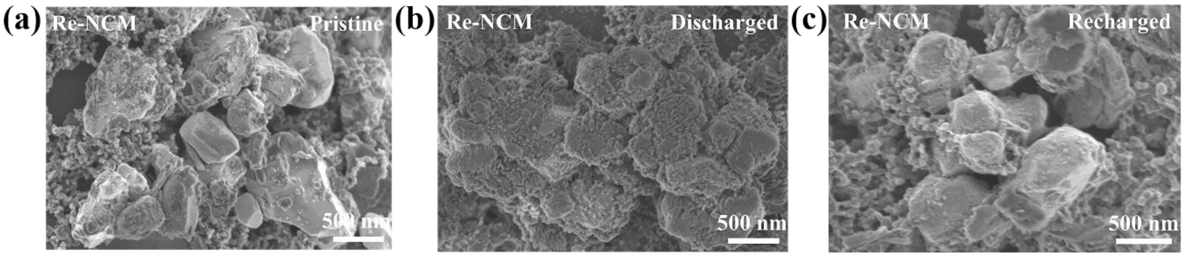


**Figure. S14.** SEM images of a) pristine, b) full discharged and c) full recharged Re-NCM cathode.

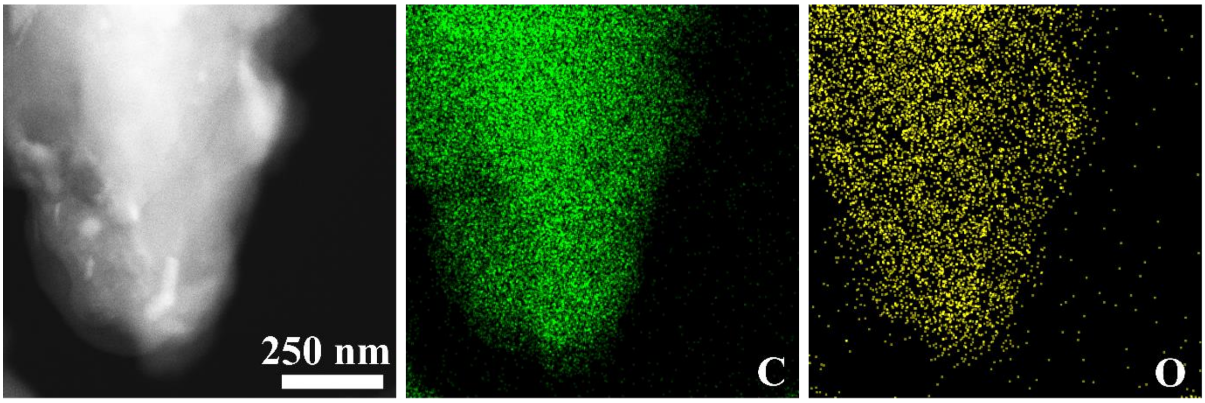


**Figure. S15.** TEM and corresponding EDX elemental mapping images of full discharged carbon-free Re-NCM cathode.

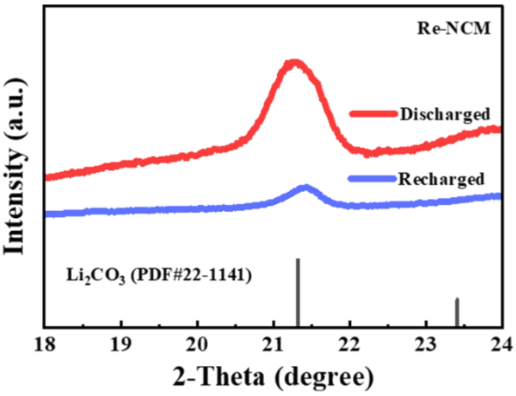


**Figure. S16.** XRD patterns of full discharged and recharged Re-NCM cathode.

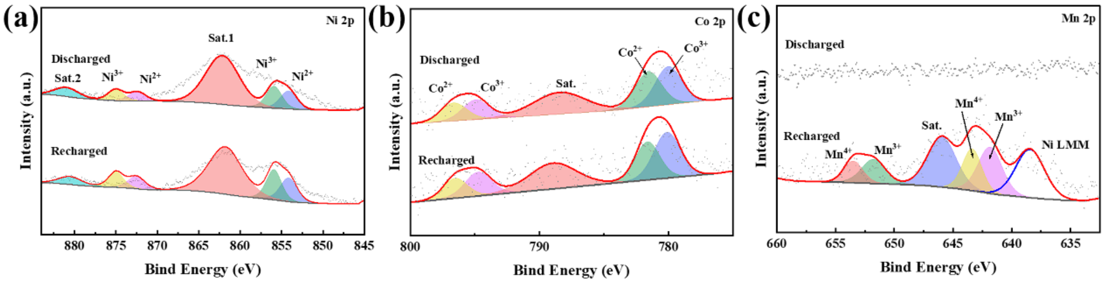


**Figure. S17.** a) Ni 2p, b) Co 2p, and c) Mn 2p XPS spectra of full discharged and recharged Re-NCM-H3 cathode.

**Table S5.** The Ohmic impedance and electron transfer resistance of Pristine, Discharged, Recharged Re-NCM-H3 cathode

| Sample | $R_1$ (ohm) | $R_2$ (ohm) |
|---|---|---|
| Pristine | 13.74 | 303.1 |
| Discharged | 11.95 | 717.3 |
| Recharged | 17.94 | 468.1 |

References

[1] J. Zhou, X. Li, C. Yang, Y. Li, K. Guo, J. Cheng, D. Yuan, C. Song, J. Lu, B. Wang, *Adv. Mater.* **2019**, *31*, 1804439.

[2] Z. Xie, X. Zhang, Z. Zhang, Z. Zhou, *Adv. Mater.* **2017**, *29*, 1605891.

[3] K. Chen, G. Huang, J. Ma, J. Wang, D. Yang, X. Yang, Y. Yu, X. Zhang, *Angew. Chem. Int. Ed.* **2020**, *59*, 16661.

[4] X. Li, H. Wang, Z. Chen, H. Xu, W. Yu, C. Liu, X. Wang, K. Zhang, K. Xie, K. P. Loh, *Adv. Mater.* **2019**, *31*, 1905879.

[5] S. Ma, H. Yao, D. Lei, X. Guo, Y. Lu, Q. Liu, Z. Li, *Chem. Commun.* **2018**, *54*, 8072.

[6] S. Li, Y. Dong, J. Zhou, Y. Liu, J. Wang, X. Gao, Y. Han, P. Qi, B. Wang, *Energy Environ. Sci.* **2018**, *11*, 1318.

[7] R. Pipes, A. Bhargav, A. Manthiram, *ACS Appl. Mater. Interfaces* **2018**, *10,* 37119.

[8] J. Chen, K. Zou, P. Ding, J. Deng, C. Zha, Y. Hu, X. Zhao, J. Wu, J. Fan, Y. Li, *Adv. Mater.* **2019**, *31*, 1805484.

[9] M. Asadi, B. Sayahpour, P. Abbasi, A. T. Ngo, K. Karis, J. R. Jokisaari, C. Liu, B. Narayanan, M. Gerard, P. Yasaei, X. Hu, A. Mukherjee, K. C. Lau, R. S. Assary, F. Khalili-Araghi, R. F. Klie, L. A. Curtiss, A. Salehi-Khojin, *Nature* **2018**, *555*, 502.

[10] Y. Qiao, S. Xu, Y. Liu, J. Dai, H. Xie, Y. Yao, X. Mu, C. Chen, D. J. Kline, E. M. Hitz, B. Liu, J. Song, P. He, M. R. Zachariah, L. Hu, *Energy Environ. Sci.* **2019**, *12*, 1100.

[11] R. Pipes, J. He, A. Bhargav, A. Manthiram, *Energy Storage Mater.* **2020**, *31*, 95.

[12] J. Lu, Y. Lee, X. Luo, K. C. Lau, M. Asadi, H.-H. Wang, S. Brombosz, J. Wen, D. Zhai, Z. Chen, D. Miller, Y. S. Jeong, J.-B. Park, Z. Z. Fang., B. Kumar, A. Salehi-Khojin, Y.-Y. Sun, L. A. Curtiss, K. Amine, *Nature* **2016**, *529*, 377.

[13] Z. Zhang, W.-L. Bai, K.-X. Wang, J.-S. Chen, *Energy Environ. Sci.* **2020**, *13*, 4717.

[14] B. Ge, Y. Sun, J. Guo, X. Yan, C. Fernandez, Q. Peng, *Small* **2019**, *15*, 1902220.
